# A Preliminary Investigation of the Anti-*Salmonella Enteritidis* Potential of Quercetin in Chickens Using Network Pharmacology, Molecular Docking, and In Vitro Antibacterial Assays

**DOI:** 10.3390/cimb48040409

**Published:** 2026-04-16

**Authors:** Qi Xiao, Yufeng Yan, Zihao Zhao, Xinyue Zhang, Tengfei Jiang, Fanzhi Kong

**Affiliations:** College of Animal Science and Veterinary Medicine, Heilongjiang Bayi Agricultural University, No. 5 Xinfeng Road, Sartu District, Daqing 163319, China; xiaoqi0401@byau.edu.cn (Q.X.); yanyufeng030314@163.com (Y.Y.); 13009073038@163.com (Z.Z.); zhangxinyue1@byau.edu.cn (X.Z.); tengfeijiang207@byau.edu.cn (T.J.)

**Keywords:** quercetin, *Salmonella Enteritidis*, network pharmacology, molecular docking

## Abstract

*Salmonella Enteritidis* is a major threat to poultry health and food safety, underscoring the need for safe alternatives to conventional antibiotics. In this study, quercetin, a natural flavonoid with antibacterial and immunomodulatory properties, was evaluated using an integrated approach combining network pharmacology, molecular docking, in vitro antibacterial assays, and preliminary in vivo validation. Potential targets of quercetin and *Salmonella Enteritidis* were identified from the TCMSP and GeneCards databases, followed by protein–protein interaction analysis, topological screening, and GO/KEGG enrichment analyses. Five core targets, namely IL1B, IL6, STAT1, PTGS2, and IFNG, were identified and were mainly enriched in immune- and inflammation-related pathways. Molecular docking suggested favorable interactions between quercetin and these predicted targets. In vitro, quercetin showed moderate antibacterial activity against *Salmonella Enteritidis*, with a minimum inhibitory concentration of 256 μg/mL and a minimum bactericidal concentration of 512 μg/mL. In vivo, quercetin alleviated intestinal histopathological damage and reduced the transcriptional expression of the five target genes in infected chicks in a dose-dependent manner, with more evident effects at doses of 512 mg/kg or higher. These findings provide preliminary evidence that quercetin may exert both direct antibacterial and host-associated protective effects against *Salmonella Enteritidis*, although the underlying mechanisms require further validation.

## 1. Introduction

*Salmonella Enteritidis* is one of the most important zoonotic pathogens in poultry production and remains a major concern for both animal health and food safety [1]. In chicks, infection can lead to acute systemic disease and elevated mortality, whereas in adult laying hens the pathogen often persists as asymptomatic intestinal colonization and may invade the reproductive tract, thereby increasing the risk of egg contamination and foodborne transmission [2]. Antibiotics have long been used to prevent or control *Salmonella* infection in poultry [3]. However, the extensive use of antimicrobials in animal production has accelerated the emergence and spread of resistant *Salmonella* strains, reducing treatment efficacy and raising concerns over the dissemination of resistance determinants through the food chain [4,5]. This situation has increased interest in safe, effective, and sustainable alternatives to traditional antibiotics.

Quercetin (3,3′,4′,5,7-pentahydroxyflavone) is a naturally occurring flavonoid that is widely distributed in fruits, vegetables, and medicinal plants [6]. Its antibacterial actions have been linked to disruption of membrane integrity, interference with energy metabolism, inhibition of biofilm formation, and modulation of virulence-associated processes [7,8,9]. In livestock and poultry research, quercetin has also been reported to influence host immune and antioxidant responses, suggesting that its anti-infective activity may involve both direct effects on pathogens and indirect effects on the host [10,11,12]. Despite these observations, the molecular basis by which quercetin may inhibit *Salmonella Enteritidis* in chickens remains insufficiently defined. In particular, the putative host targets, biological pathways, and integrated mechanism underlying its anti-*Salmonella* activity have not been systematically clarified. Network pharmacology provides a useful framework for addressing such questions because it enables the analysis of drug-target-disease relationships at a systems level. When combined with molecular docking and experimental verification, this strategy can help identify potential core targets and pathways relevant to the pharmacological action of natural compounds [13,14]. In the present study, we used an integrated approach combining network pharmacology, molecular docking, in vitro antibacterial assays, and preliminary in vivo validation to investigate the potential anti-*Salmonella Enteritidis* activity of quercetin in chickens. Specifically, we aimed to identify predicted host-associated targets and pathways of quercetin, assess the potential interactions between quercetin and these targets through molecular docking, determine its direct antibacterial activity against *Salmonella Enteritidis* in vitro, and provide preliminary in vivo support through intestinal histopathology and transcriptional analysis of selected target genes in infected chicks. Given the current level of evidence, this study was designed to provide a preliminary framework for understanding the possible antibacterial and host-associated protective effects of quercetin rather than definitive proof of mechanism or in vivo efficacy. The findings may therefore offer a basis for further evaluation of quercetin as a candidate natural compound for the control of *Salmonella Enteritidis* in poultry.

## 2. Materials and Methods

### 2.1. Collection of Quercetin-Related Targets

Potential targets associated with quercetin were retrieved from the Traditional Chinese Medicine Systems Pharmacology Database and Analysis Platform (TCMSP; https://tcmsp-e.com/tcmspsearch.php (accessed on 10 August 2025)) using the keyword “quercetin” in the Chemical name field. After removal of duplicate and invalid entries, the resulting target set was retained for subsequent analysis.

### 2.2. Collection of Salmonella Enteritidis-Related Targets

Targets related to *Salmonella Enteritidis* were retrieved from the GeneCards database (https://www.genecards.org) using the keyword “*Salmonella Enteritidis*”. Duplicate entries and targets with low relevance were excluded prior to intersection analysis.

### 2.3. Identification of Overlapping Targets

The quercetin-related target set and the *Salmonella Enteritidis*-related target set were imported into the Venny 2.1.0 online tool (https://bioinfogp.cnb.csic.es/tools/venny/ (accessed on 15 August 2025)) to identify overlapping targets. The shared targets were considered potential targets through which quercetin may exert inhibitory effects against *Salmonella Enteritidis*.

### 2.4. Construction of the Quercetin-Target Network

A network file containing quercetin-related targets and a type file containing the overlapping targets were prepared in Microsoft Excel and imported into Cytoscape 3.10.3 for network construction and visualization. Network topology was adjusted according to node degree values.

### 2.5. Protein–Protein Interaction Analysis and Core Target Screening

The overlapping targets were submitted to the STRING database (https://cn.string-db.org/) using the “Multiple proteins” option, with the species set to *Gallus gallus*. The resulting protein–protein interaction (PPI) network was exported in TSV format and analyzed in Cytoscape. Using the Network Analyzer tool (Cytoscape 3.9.1), degree centrality, betweenness centrality, and closeness centrality were calculated for each node. Targets with all three parameters above the network average were defined as core targets.

### 2.6. GO and KEGG Enrichment Analyses

The identified core targets were submitted to the DAVID database (https://davidbioinformatics.nih.gov/ (accessed on 16 August 2025)) for Gene Ontology (GO) enrichment and Kyoto Encyclopedia of Genes and Genomes (KEGG) pathway analyses. OFFICIAL_GENE_SYMBOL was used as the gene identifier and *Gallus gallus* as the species. The enriched terms were visualized using the Microbioinformatics online platform (http://www.bioinformatics.com.cn/), with *p* ≤ 0.01 considered statistically significant.

### 2.7. Molecular Docking

To assess the potential interaction between quercetin and the core target proteins, molecular docking was performed. Three-dimensional structures of the core proteins were downloaded from the RCSB PDB database (https://www.rcsb.org/), and the three-dimensional structure of quercetin was obtained from TCMSP. Protein structures were preprocessed in PyMOL 3.1.0 by removing water molecules and separating original ligands where necessary. Docking simulations were then performed using AutoDock 1.5.7 after hydrogen addition, charge assignment, and grid box configuration. Binding energies were recorded, and the docking conformations were visualized in PyMOL after file conversion with Open Babel. The target-screening and enrichment analyses were performed using *Gallus gallus* as the reference species. For molecular docking, the protein models used for each target were obtained from the RCSB PDB database IL1B (PDB ID: 1ILR), IL6 (PDB ID: 1ALU), STAT1 (PDB ID: 1BGF), PTGS2 (PDB ID: 3NT1), and IFNG (PDB ID: 4EQ2), and the docking results were interpreted only as predictions of possible binding interactions.

### 2.8. In Vitro Antibacterial Assay

#### 2.8.1. Materials

The *Salmonella Enteritidis* standard strain CICC21513 was purchased from the China Center of Industrial Culture Collection (CICC). Quercetin (purity ≥ 98%) was preserved in the Laboratory of Veterinary Pharmacology and Toxicology, College of Animal Science and Technology, Heilongjiang Bayi Agricultural University. LB broth (Beijing, China, Coolarber, PM0010), Mueller-Hinton medium (Beijing, China, Coolarber, PM0530), Nutrient agar (Beijing, China, Coolarber, MM1891), DMSO (Beijing, China, Coolarber, CD4731C), Tween 80 (Beijing, China, Coolarber, CTT11581), and PEG400 (Beijing, China, Coolarber, CP8187). Sterile 96-well plates (Wuxi, China, NEST, 514201) were used throughout the assays.

#### 2.8.2. Determination of MIC and MBC

Quercetin was dissolved in a mixed solvent of DMSO, Tween 80, and PEG400 (1:1:1, *v*/*v*/*v*) and sterilized through a 0.22 μm filter to prepare the stock solution. The final concentration of each solvent in the stock solution was 2.5%. The *Salmonella Enteritidis* strain was activated in LB broth at 37 °C with shaking at 180 r/min to the logarithmic growth phase, and the bacterial suspension was adjusted to approximately 1.5 × 10^6^ CFU/mL using sterile saline. For MIC determination, the broth microdilution method was used. Quercetin was subjected to two-fold serial dilution in MH medium to generate concentrations ranging from 512 to 0.125 μg/mL. Aliquots of the bacterial suspension were added to each dilution. Negative control wells contained bacterial suspension with culture medium and the corresponding solvent system without quercetin; blank control wells contained sterile medium only; and the positive control contained 10% Enrofloxacin solution. After incubation at 37 °C for 18–24 h, bacterial growth was assessed visually and by measuring optical density at 600 nm. The MIC was defined as the lowest concentration at which no visible bacterial growth was observed. For MBC determination, aliquots from wells or cultures showing no visible growth at the MIC and higher concentrations were plated onto nutrient agar and incubated at 37 °C for 18–24 h. The MBC was defined as the lowest concentration reducing the initial viable count by more than 99.9%. All assays were performed in triplicate in three independent experiments.

### 2.9. In Vivo Therapeutic Experiment of Quercetin

#### 2.9.1. Animals and Treatments

A total of 60 one-day-old white-feathered broiler chicks (purchased from Zhanshan farm, Harbin, China) were used in the in vivo experiment. After a 3-day acclimation period with ad libitum access to feed and water, all chicks were confirmed to be *Salmonella*-negative by fecal culture and then randomly assigned to six groups: a normal control group, an infected untreated group, an antibiotic-treated group, a quercetin high-dose group, a quercetin medium-dose group, and a quercetin low-dose group. Chicks in the infected groups were orally challenged once with 0.2 mL of *Salmonella Enteritidis* suspension at 1.5 × 10^7^ CFU/mL using a sterile metal gavage needle, whereas chicks in the normal control group received an equal volume of sterile PBS. After challenge, the animals were monitored daily for general condition, feed intake, fecal characteristics, and mortality. Treatment was initiated 24 h after infection and continued for 5 consecutive days. Enrofloxacin was administered in drinking water at 0.5 g/L and freshly prepared each day. Quercetin was mixed into the feed at doses of 1024, 512, and 256 mg/kg for the high-, medium-, and low-dose groups, respectively, while the chicks were allowed free access to normal drinking water. Feed and drinking water were renewed daily to ensure the stability of the treatments. All animal experiments were approved by the Animal Experiment Ethical Committee of Heilongjiang Bayi Agricultural University (Permit Number: DWKJXY2025099) in accordance with the Regulations for the Administration of Affairs Concerning Experimental Animals.

#### 2.9.2. Hematoxylin and Eosin (H&E) Staining of Intestinal Tissue Sections

On day 6 post-infection, two chicks were randomly selected from each group for histopathological examination. Jejunum and ileum tissues were collected, fixed, embedded in paraffin, sectioned, and stained with H&E according to standard procedures. Briefly, paraffin sections were deparaffinized in xylene, rehydrated through a graded ethanol series, stained with hematoxylin, differentiated in 1% hydrochloric acid solution, blued in 0.6–0.7% ammonia water, counterstained with eosin, dehydrated, cleared, and mounted with neutral resin. Histological changes were observed under a light microscope.

#### 2.9.3. Real-Time Quantitative PCR (RT-qPCR)

The RT-qPCR reaction system was prepared according to the manufacturer’s instructions for TB Green Premix Ex Taq (Takara, Beijing, China). The total reaction volume was 20 μL. GAPDH was used as the internal reference gene. Each sample was analyzed with three technical replicates, and relative gene expression was calculated using the 2^−ΔΔCt^ method. The primer sequences are listed in Table 1.

#### 2.9.4. Statistical Analysis

In this study, statistical analyses were performed using GraphPad Prism 11.0 software, and all data are presented as the mean ± standard deviation (Mean ± SD). One-way analysis of variance (one-way ANOVA) and Student’s *t*-test were used for statistical comparisons, as appropriate. Statistical significance was defined as follows: * *p* < 0.05; ** *p* < 0.01; *** *p* < 0.001. Differences were considered statistically significant when *p* < 0.05.

## 3. Results

### 3.1. Acquisition of Quercetin-Related Targets

**Figure 1:**
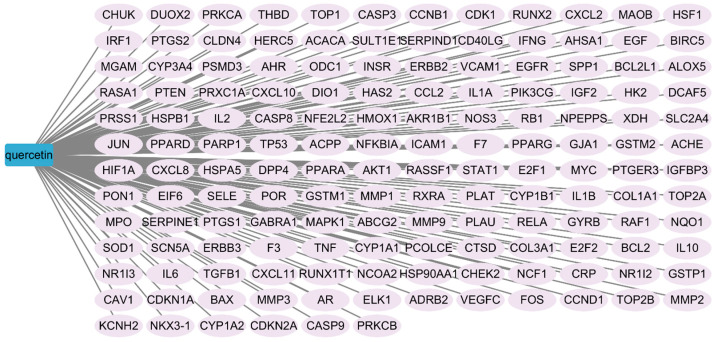
Network of quercetin-related targets.

Through the retrieval and screening of the TCMSP database, a total of 150 valid quercetin-related targets were finally obtained after removing duplicate and invalid targets (Figure 1). All the obtained targets were used as the research basis for the subsequent intersection analysis with *Salmonella* *Enteritidis*-related targets.

### 3.2. Identification of Quercetin-Related and Overlapping Targets

**Figure 2:**
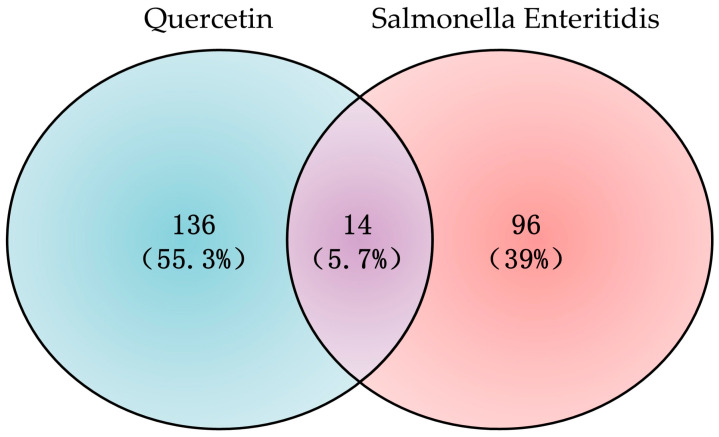
Venn diagram of overlapping targets between quercetin and *Salmonella Enteritidis*.

After database retrieval and data cleaning, 150 valid quercetin-related targets and 110 *Salmonella Enteritidis*-related targets were retained. Venn analysis identified 14 overlapping targets (Figure 2), which were considered potential targets of quercetin against *Salmonella Enteritidis*.

### 3.3. PPI Network Construction and Core Target Screening

**Figure 3:**
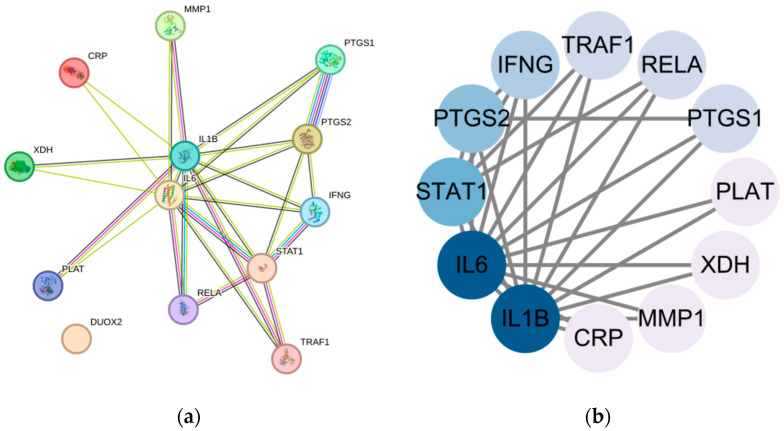
Protein–protein interaction (PPI) networks of quercetin and *Salmonella Enteritidis* shared targets. (**a**) PPI network of the 14 potential targets; (**b**) Core target network constructed from topological analysis, highlighting five key nodes: IL1B, IL6, STAT1, PTGS2, and IFNG.

The 14 overlapping targets were submitted to STRING to construct the PPI network (Figure 3a). Topological analysis in Cytoscape identified five core targets (Figure 3b), namely IL1B, IL6, STAT1, PTGS2, and IFNG (Table 2). These genes occupied central positions in the interaction network, suggesting that they may play important roles in the anti-*Salmonella Enteritidis* activity of quercetin.

### 3.4. GO Enrichment Analysis

**Figure 4:**
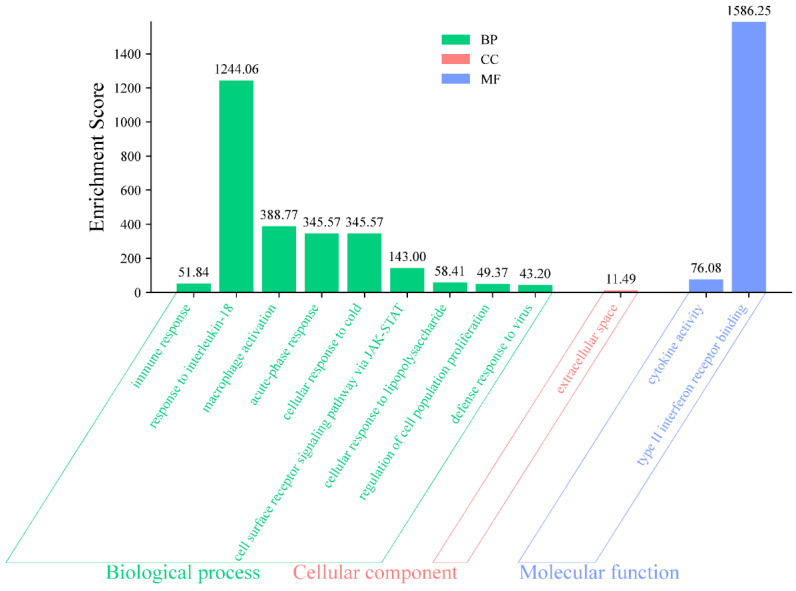
GO enrichment analysis of core targets. Significantly enriched terms are shown for Biological Process (BP), Cellular Component (CC), and Molecular Function (MF). The analysis was performed using the DAVID database with *Gallus gallus* as the reference species.

GO enrichment analysis of the five core targets identified significantly enriched terms in biological process, cellular component, and molecular function categories. The enriched biological processes were mainly associated with immune and inflammatory regulation, including response to interleukin-18, immune response, macrophage activation, and acute-phase response. The most prominent cellular component term was extracellular space, while the major molecular function terms included cytokine activity and type II interferon receptor binding (Figure 4). These results indicate that the putative activity of quercetin is closely related to modulation of host immune–inflammatory responses.

### 3.5. KEGG Pathway Enrichment Analysis

**Figure 5:**
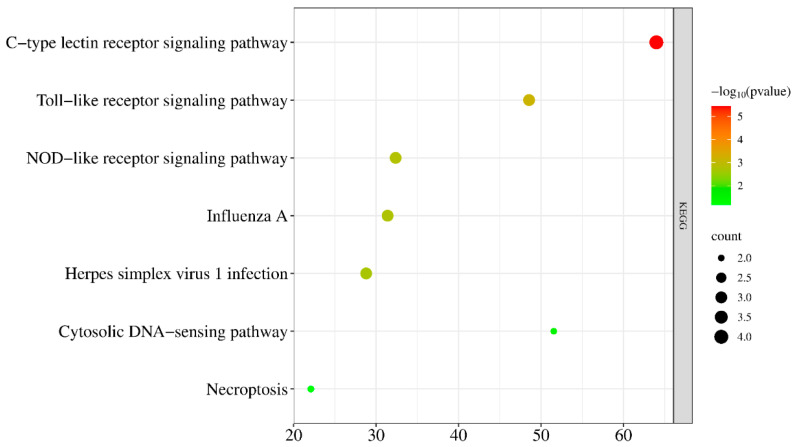
KEGG pathway enrichment analysis of core targets (the bubble plot illustrates significantly enriched signaling pathways).

KEGG enrichment analysis showed that the core targets were mainly involved in pathways related to innate immune recognition and inflammatory signaling, including the Toll-like receptor, NOD-like receptor, and C-type lectin receptor signaling pathways. Additional enrichment was observed in pathways associated with pathogen response and cell death regulation, such as necroptosis and the cytosolic DNA-sensing pathway (Figure 5). These findings suggest that quercetin may influence the host response to *Salmonella Enteritidis* by regulating key nodes of pathogen recognition and downstream inflammatory signaling.

### 3.6. Molecular Docking Results

**Figure 6:**
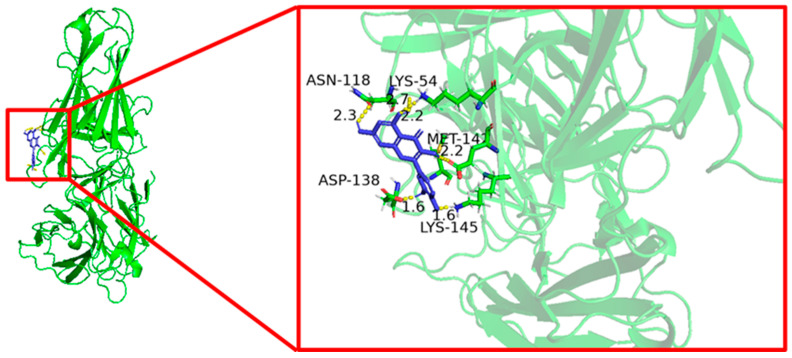
Molecular docking of quercetin with IL1B.

**Figure 7:**
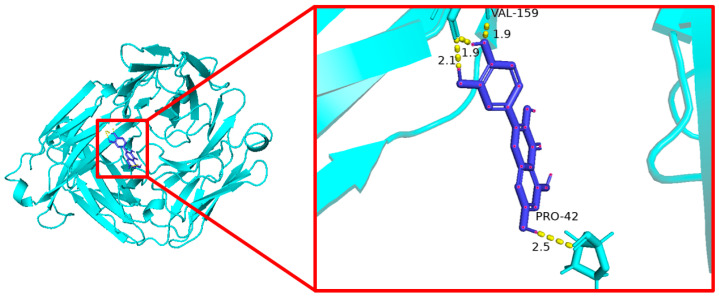
Molecular docking of quercetin with IL6.

**Figure 8:**
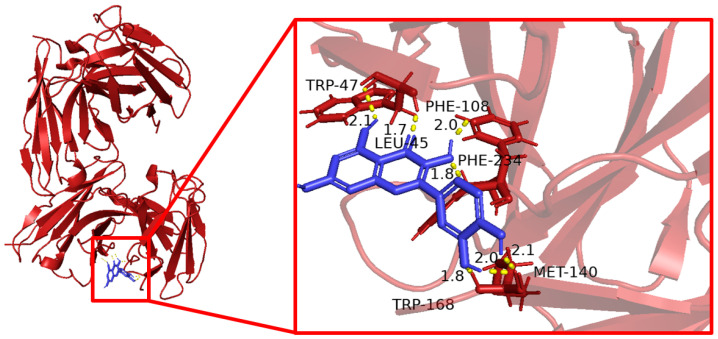
Molecular docking of quercetin with IFNG.

**Figure 9:**
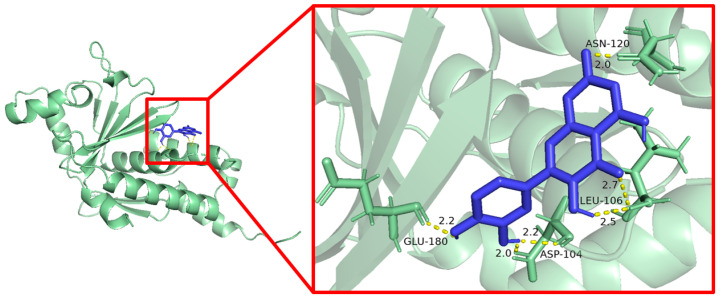
Molecular docking of quercetin with STAT1.

**Figure 10:**
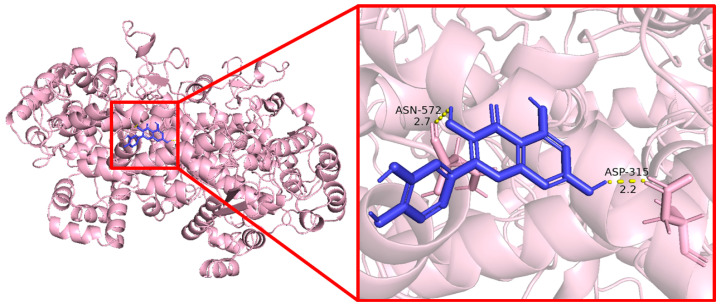
Molecular docking of quercetin with PTGS2.

Molecular docking was used to evaluate the interaction between quercetin and the five core target proteins. Quercetin showed favorable binding to IL1B, IL6, STAT1, PTGS2, and IFNG (Figure 6, Figure 7, Figure 8, Figure 9 and Figure 10), and stable hydrogen bond interactions were observed in the predicted docking conformations. The hydrogen bond distances were within a reasonable range, supporting the possibility that quercetin can interact directly with these proteins.

### 3.7. In Vitro Antibacterial Activity of Quercetin

**Figure 11:**
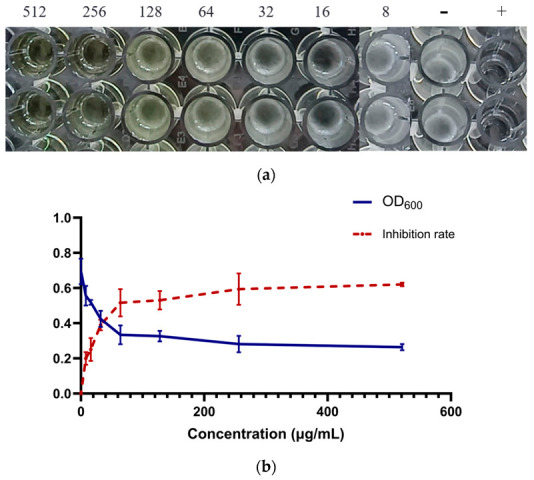
In vitro antibacterial activity of quercetin against *Salmonella Enteritidis*. (**a**) MIC determination in a 96-well plate. Clear wells indicate inhibition of bacterial growth. (**b**) Inhibition rate curve showing dose-dependent antibacterial effect.

**Figure 12:**
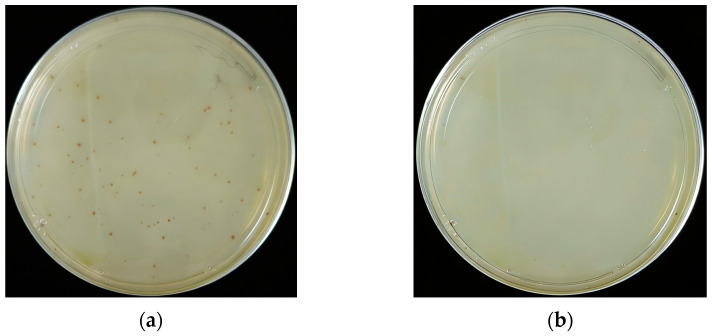
Minimum bactericidal concentration (MBC) of quercetin. (**a**) Plate culture from 256 μg/mL quercetin treatment showing partial bacterial growth; (**b**) plate culture from 512 μg/mL quercetin treatment showing no visible bacterial colonies, indicating bactericidal activity.

The broth microdilution assay showed that quercetin inhibited the growth of *Salmonella Enteritidis* in vitro, with an MIC of 256 μg/mL (Figure 11) and an MBC of 512 μg/mL (Figure 12). After subculture of aliquots from the clear wells, no visible bacterial colonies were observed at 512 μg/mL, confirming the bactericidal concentration under the tested conditions. It was found that when the quercetin concentration reached 256 μg/mL, there was no obvious bacterial growth, which demonstrates that the minimum inhibitory concentration (MIC) of quercetin is 256 μg/mL. The wells containing quercetin at concentrations of 256 μg/mL or higher remained clear after incubation, whereas obvious turbidity was observed in the negative control. The positive drug control also showed complete growth inhibition. These results confirm that quercetin possesses direct antibacterial and bactericidal activity against *Salmonella Enteritidis* under the conditions tested.

### 3.8. In Vivo Effects of Quercetin on Intestinal Tissue Histopathology

**Figure 13:**
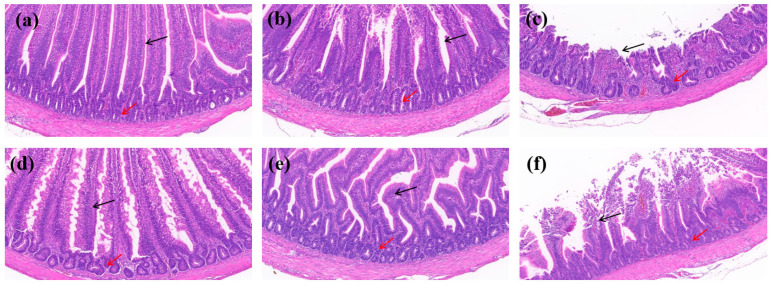
Representative H&E-stained images of jejunal tissues from different treatment groups (original magnification, ×20). (**a**) Control group; (**b**) enrofloxacin group; (**c**) infected untreated group; (**d**) quercetin high-dose group; (**e**) quercetin medium-dose group; and (**f**) quercetin low-dose group. Black arrows indicate villus damage, including villus necrosis, fragmentation, or structural disruption, whereas red arrows indicate inflammatory cell infiltration and crypt abnormalities.

**Figure 14:**
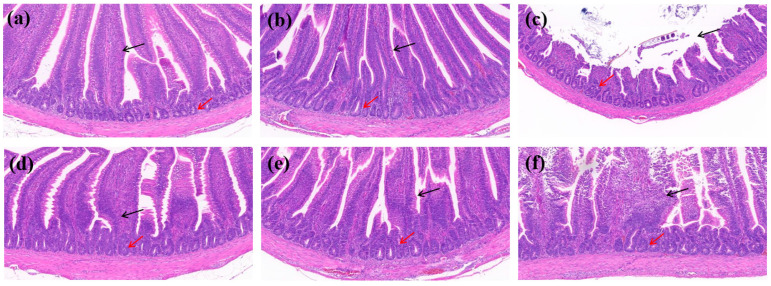
Representative H&E-stained images of ileal tissues from different treatment groups (original magnification, ×20). (**a**) Control group; (**b**) enrofloxacin group; (**c**) infected untreated group; (**d**) quercetin high-dose group; (**e**) quercetin medium-dose group; and (**f**) quercetin low-dose group. Black arrows indicate villus damage, including villus necrosis, fragmentation, or structural disruption, whereas red arrows indicate inflammatory cell infiltration and crypt abnormalities.

H&E staining showed that the infected untreated group exhibited significant intestinal histopathological damage compared with the control group, including villus necrosis and fragmentation, partial structural loss, disorganization of vascular loop structures, inflammatory cell infiltration, and a reduced number of crypts with mild crypt atypia. The quercetin low-dose group still showed mild histopathological abnormalities, including slight villus structural disruption and local crypt damage, although no obvious inflammatory cell infiltration was observed and the submucosa and muscularis propria remained intact. In contrast, the enrofloxacin group and the quercetin medium- and high-dose groups displayed generally preserved intestinal morphology, with intact villous epithelial structure, normal crypt architecture, and no obvious inflammatory infiltration or tissue necrosis. These findings suggest that quercetin at doses of 512 mg/kg or higher alleviated intestinal tissue damage caused by *Salmonella Enteritidis* infection. Detailed histopathological changes are shown in Figure 13 and Figure 14.

### 3.9. RT-qPCR Analysis of Intestinal Tissues

**Figure 15:**
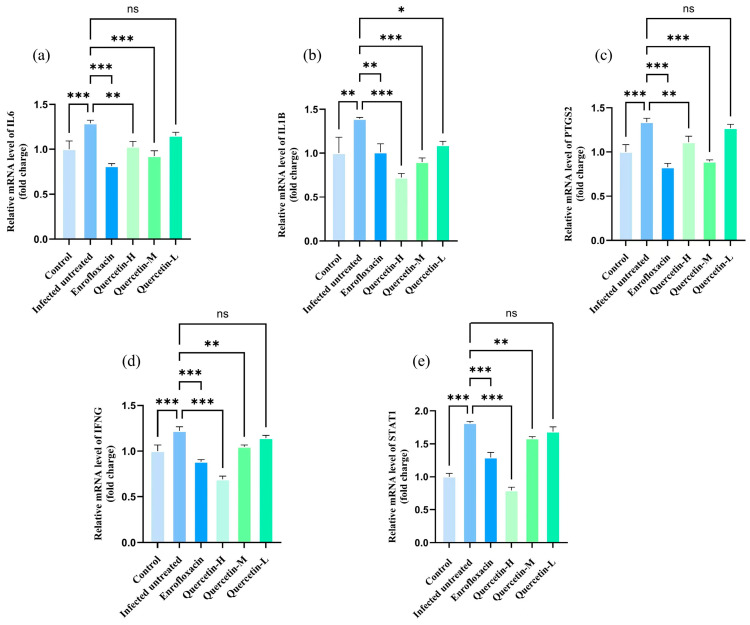
RT-qPCR analysis of target gene expression in chick intestinal tissues. (**a**) IL6 mRNA expression; (**b**) IL1β mRNA expression; (**c**) PTGS2 mRNA expression; (**d**) IFNγ mRNA expression; and (**e**) STAT1 mRNA expression. Data are presented as mean ± SEM (n = 3). Statistical significance was analyzed by one-way ANOVA or Student’s *t*-test, as appropriate. * *p* < 0.05, ** *p* < 0.01, *** *p* < 0.001.

RT-qPCR analysis showed that the mRNA expression levels of IL6, IL1β, PTGS2, IFNγ, and STAT1 were significantly increased in the intestinal tissues of the infected untreated group compared with the control group, indicating that *Salmonella Enteritidis* infection induced a marked local inflammatory response (Figure 15). Quercetin treatment reduced the expression of these genes in a dose-dependent manner. Among the quercetin-treated groups, the high-dose group showed the strongest inhibitory effect, whereas the medium-dose group also showed clear downregulation, although to a lesser extent. The low-dose group showed a weaker reduction than the enrofloxacin group. Overall, these results indicate that quercetin attenuated the transcriptional upregulation of inflammation-related genes induced by *Salmonella Enteritidis*, and this trend was generally consistent with the predicted targets identified by the network pharmacology analysis.

## 4. Discussion

In this study, we used an integrated strategy combining network pharmacology, molecular docking, in vitro antibacterial assays, and preliminary in vivo validation to explore the potential anti-*Salmonella Enteritidis* activity of quercetin. Five core targets, namely IL1B, IL6, STAT1, PTGS2, and IFNG, were identified by network analysis, and enrichment analysis suggested that these targets were mainly associated with immune- and inflammation-related biological processes and signaling pathways. At the same time, the broth microdilution assay showed that quercetin exerted direct in vitro antibacterial activity against *Salmonella Enteritidis*, with an MIC of 256 μg/mL and an MBC of 512 μg/mL. In addition, the newly added in vivo results showed that quercetin alleviated intestinal histopathological injury and reduced the transcriptional expression of the five predicted core targets in infected chicks in a dose-dependent manner. Taken together, these findings suggest that quercetin may have both host-associated and direct antibacterial relevance; however, the present evidence should still be interpreted cautiously because the host-related mechanisms remain only partially validated.

The host response to *Salmonella Enteritidis* infection is shaped not only by bacterial colonization and invasion but also by the magnitude and quality of inflammatory signaling. Excessive production of inflammatory mediators may aggravate intestinal barrier injury and contribute to disease progression [15,16,17,18,19]. In this context, the identification of IL1B, IL6, STAT1, PTGS2, and IFNG as predicted core targets is biologically plausible, because these molecules are closely involved in cytokine signaling, inflammatory regulation, and antimicrobial immunity [17,18,19]. Similarly, KEGG enrichment highlighted pathways such as Toll-like receptor, NOD-like receptor, and C-type lectin receptor signaling, which are important in innate immune recognition of invading pathogens [20,21,22]. Importantly, the RT-qPCR results obtained from intestinal tissues of infected chicks showed that these five genes were markedly upregulated after infection and were reduced following quercetin treatment in a dose-dependent manner, with the strongest effect observed in the high-dose group. This pattern was generally consistent with the network pharmacology prediction and provides preliminary in vivo transcriptional support for the proposed host-associated targets. Nevertheless, these data should still be regarded as supportive rather than definitive, because the present study does not establish direct target engagement or fully define the upstream regulatory events by which quercetin modulates these pathways in infected tissues.

The molecular docking analysis provided additional in silico support for possible interactions between quercetin and the five predicted core proteins. Nevertheless, docking results only indicate potentially favorable binding conformations and do not establish target engagement, biological activity, or pharmacological relevance under physiological conditions. This point is particularly important here because the identified targets are primarily host immune–inflammatory molecules rather than bacterial molecular targets. Therefore, the docking results should not be interpreted as direct evidence of the antibacterial mechanism of quercetin against *Salmonella Enteritidis*, but rather as supportive information consistent with the network-based predictions and the subsequent transcriptional observations in vivo.

The antibacterial assay demonstrated that quercetin inhibited *Salmonella Enteritidis* growth in vitro, with an MIC of 256 μg/mL and an MBC of 512 μg/mL under the tested conditions. These values suggest a moderate antibacterial effect rather than a highly potent bactericidal activity. This interpretation is more consistent with the current literature, in which quercetin has been reported to exert antimicrobial, antibiofilm, and anti-virulence effects against a range of bacterial species, including Salmonella, but often with mechanisms that remain incompletely defined [11,12,23,24,25,26]. Accordingly, the in vitro results obtained here support the view that quercetin has measurable direct activity against *Salmonella Enteritidis*, but they do not by themselves clarify whether this activity is mediated by membrane disruption, metabolic interference, virulence suppression, or other bacterial processes.

Notably, the newly added histopathological data further strengthen the biological relevance of the present findings. H&E staining showed that *Salmonella Enteritidis* infection caused marked intestinal injury, including villus necrosis and fragmentation, crypt damage, and inflammatory cell infiltration, whereas these lesions were alleviated in the enrofloxacin group and in the quercetin medium- and high-dose groups. In particular, quercetin at 512 mg/kg or higher was associated with better preservation of villus and crypt architecture, suggesting a protective or reparative effect against infection-induced intestinal damage. When considered together with the RT-qPCR results, these observations indicate that quercetin may alleviate intestinal injury not only through moderate direct antibacterial activity but also through attenuation of excessive host inflammatory responses. At the present stage, however, the in silico, in vitro, and in vivo findings should still be viewed as complementary rather than fully mechanistically integrated evidence.

Several limitations should therefore be acknowledged. First, the antibacterial assay was performed using a standard strain only, and the activity of quercetin against clinical isolates or multidrug-resistant *Salmonella Enteritidis* strains remains unknown. Second, although the added intestinal histopathology and RT-qPCR analyses provided preliminary in vivo support for the predicted host-associated targets, the identified pathways still require further validation at the protein and functional levels in relevant chicken cells or tissues. Third, the docking results do not confirm biological activity and are further limited by the availability and source of structural models. Fourth, the current antimicrobial assay was limited to endpoint growth inhibition and did not address bacterial cellular mechanisms, virulence traits, host–pathogen interaction, or physiologically relevant gastrointestinal conditions. Finally, the in vivo efficacy, pharmacokinetics, optimal dosage, and practical applicability of quercetin in poultry production remain to be determined. Future studies should therefore focus on protein-level validation of the predicted host targets, mechanistic investigation of bacterial responses to quercetin, and more comprehensive evaluation in animal infection models.

## 5. Conclusions

In conclusion, quercetin showed moderate in vitro antibacterial activity against *Salmonella Enteritidis* and was associated, through network pharmacology and molecular docking analyses, with several predicted host immune–inflammatory targets, including IL1B, IL6, STAT1, PTGS2, and IFNG. In addition, the newly added in vivo results showed that quercetin alleviated intestinal histopathological damage and reduced the transcriptional expression of these inflammation-related genes in infected chicks in a dose-dependent manner, with more evident effects observed at doses of 512 mg/kg or higher. Taken together, these findings suggest that quercetin may have anti-*Salmonella Enteritidis* potential at both the bacterial and host-associated levels. However, the proposed host-related mechanisms remain preliminary, and the current evidence should still be interpreted with caution. Further studies at the protein, functional, and in vivo efficacy levels are required to clarify the precise mechanisms and practical value of quercetin for *Salmonella Enteritidis* control in poultry.

## Figures and Tables

**Table 1:** Primers used in this study.

Primer Name	Primer Sequence (5′-3′)
IL-1β-F	AGGCTCAACATTGCGCTGTA
IL-1β-R	CTTGTAGCCCTTGATGCCCA
IL-6-F	GCTTCGACGAGGAGAGAAATGC
IL-6-R	GCCAGGTGTGTGTTTGTGTGTA
IFNγ-F	TGGCGTGAAGAGGGTGAAAGA
IFNγ-R	TCTGAGACTGGCTCCTTTTCCT
STAT1-F	CCCAAAGGACCTCACAGTCA
STAT1-R	TTACTTGATGAAGGCGCCCG
PTGS2-F	CTGCTCCCCCCCATGTCAGA
PTGS2-R	CACGTGAAGAATTCCGTGTGTT
GAPDH-F	CCACAACATACTCAGCACCTGC
GAPDH-R	GTCCTCTGGCAAAGTCCAAG

**Table 2:** Core targets of quercetin against *Salmonella Enteritidis*.

Target Name	Gene ID
Interleukin 1 Beta	IL1B
Interleukin 6	IL6
Signal Transducer and Activator of Transcription 1	STAT1
Prostaglandin-Endoperoxide Synthase 2	PTGS2
Interferon Gamma	IFNG

## Data Availability

The original contributions presented in this study are included in the article. Further inquiries can be directed to the corresponding author.
